# Pomegranate for Prevention and Treatment of Cancer: An Update

**DOI:** 10.3390/molecules22010177

**Published:** 2017-01-24

**Authors:** Pooja Sharma, Sarah F. McClees, Farrukh Afaq

**Affiliations:** 1 Department of Dermatology, University of Alabama at Birmingham, Birmingham, AL 35294, USA; vaidp@uab.edu (P.S.); smcclees@uab.edu (S.F.M.); 2 Comprehensive Cancer Center, University of Alabama at Birmingham, Birmingham, AL 35294, USA

**Keywords:** pomegranate, cancer, cell proliferation, inflammation, angiogenesis, apoptosis

## Abstract

Cancer is the second leading cause of death in the United States, and those who survive cancer may experience lasting difficulties, including treatment side effects, as well as physical, cognitive, and psychosocial struggles. Naturally-occurring agents from dietary fruits and vegetables have received considerable attention for the prevention and treatment of cancers. These natural agents are safe and cost efficient in contrast to expensive chemotherapeutic agents, which may induce significant side effects. The pomegranate (*Punica granatum* L.) fruit has been used for the prevention and treatment of a multitude of diseases and ailments for centuries in ancient cultures. Pomegranate exhibits strong antioxidant activity and is a rich source of anthocyanins, ellagitannins, and hydrolysable tannins. Studies have shown that the pomegranate fruit as well as its juice, extract, and oil exert anti-inflammatory, anti-proliferative, and anti-tumorigenic properties by modulating multiple signaling pathways, which suggest its use as a promising chemopreventive/chemotherapeutic agent. This review summarizes preclinical and clinical studies highlighting the role of pomegranate in prevention and treatment of skin, breast, prostate, lung, and colon cancers.

## 1. Introduction

Cancer is a disease of unrestricted cell proliferation. Normally considered a disease of genetic origin, research over the last several decades has established beyond doubt that various epigenetic/environmental factors play an important role in the development and/or progression of cancer [1,2]. With no single defined cause and a number of risk factors, including smoking, alcohol consumption, poor diet, and obesity, etc., cancer is widely accepted as a lifestyle disease [3,4]. Almost 1,685,210 new cancer cases will be detected in 2016 in the United States alone, and nearly 595,690 people will die of cancer [5]. Despite the considerable advancement in treatment options, the incidence and mortality from cancer continues to increase [3,5]. It is estimated that there will be virtually 20 million cancer patients by the year 2025 [5]. Therefore, attention is being focused on prevention as an ultimate strategy for the management of cancer [6,7]. It is currently estimated that two-thirds of cancer-related deaths may well be prevented through lifestyle variation, mostly through dietary means [7,8].

Almost 2500 years ago, Hippocrates recognized the importance of food for overall health. Medicinal systems across different cultures have professed and promoted the use of edible substances, especially those derived from plants, to prevent/treat diseases over last several centuries, hence, creating awareness of the potential of natural agents also known as phytochemicals as cancer chemopreventive/chemotherapeutic agents. As of today almost 47% of the available anticancer drugs in the market are derivatives of natural products or natural product mimics [9]. There is a great deal of scientific evidence to show that daily consumption of a diet rich in fruits and vegetables reduces the risk of cancer [10,11,12] and, in recent years, considerable interest has been focused on plant foods containing polyphenolic compounds [13,14]. Reports suggest that, except for 5%–10% of all cancer cases, the remaining 90%–95% are caused by environment and lifestyle [15]. So far, nearly 25,000 different phytochemicals have been identified in fruits and vegetables that have tremendous anticancer properties [15]. These phytochemicals are nontoxic and generally target multiple signaling pathways [6,14]. 

Pomegranate (*Punica granatum* L.) is obtained from a deciduous tree belonging to the family Lythraceae. There are reports that indicate that it first originated in modern day from Iran and has been cultivated through the Mediterranean region and Northern India since ancient times [16]. Today, it is cultivated in North Africa and tropical Africa, North and South America, and even in Europe for its fruit crop and also as decorative trees and shrubs. Pomegranate fruit is a rounded berry with a thick reddish skin covering approximately 200–1400 white to deep red or purple seeds. Pomegranate seeds are edible and hold strong antioxidant and anti-inflammatory properties due to their high content of hydrolysable tannins and anthocyanins [17]. As compared to the antioxidant activity of vitamin E, β-carotene, and ascorbic acid, the pomegranate antioxidants appear unique due to combinations of a wide array of polyphenols, having a broader range of action against several types of free radicals [18]. As compared to the recognized antioxidants in red wine and green tea, anthocyanins from pomegranate fruit possess significantly higher antioxidant activity [19].

Pomegranate has been used in various medicinal systems of medicine for the treatment and therapy of a multitude of diseases and ailments. In the ancient Indian medicinal system, i.e., in Ayurvedic medicine, the pomegranate was considered to be a whole pharmacy unto itself. It was recommended to be used as an antiparasitic agent and to treat diarrhea and ulcers [20,21]. The Unani system of medicine, which is another traditional system of medicine, recognizes the importance of pomegranate in the treatment of diabetes [22]. The medicinal properties of pomegranate have sparked significant interest in today’s scientific community as evidenced by the scientific research relating to health benefits of pomegranate that have been published in last few decades [14,23]. Remarkably, it is not just the pomegranate fruit itself, but other parts of the plant as well, including the bark, leaves, and roots of the pomegranate tree, that are rich in molecular constituents with therapeutic properties [21,24].

Studies have shown that pomegranate and its constituents can efficiently affect multiple signaling pathways involved in inflammation, cellular transformation, hyperproliferation, angiogenesis, initiation of tumorigenesis, and eventually suppressing the final steps of tumorigenesis and metastasis [14,23]. The pomegranate constituents are shown to modulate transcription factors, pro-apoptotic proteins, anti-apoptotic proteins, cell cycle regulator molecules, protein kinases, cell adhesion molecules, pro-inflammatory mediators, and growth factors in various cancers (Table 1). In this review article, we first discussed some of the polyphenolic constituents and mineral ions present in pomegranate, and we then discussed studies on chemopreventive/chemotherapeutic properties of pomegranate against different types of cancer, such as skin, breast, prostate, colon, and lung cancers in cell culture systems, animal models and humans.

## 2. Pomegranate Chemical Constituents

**Figure 1:**
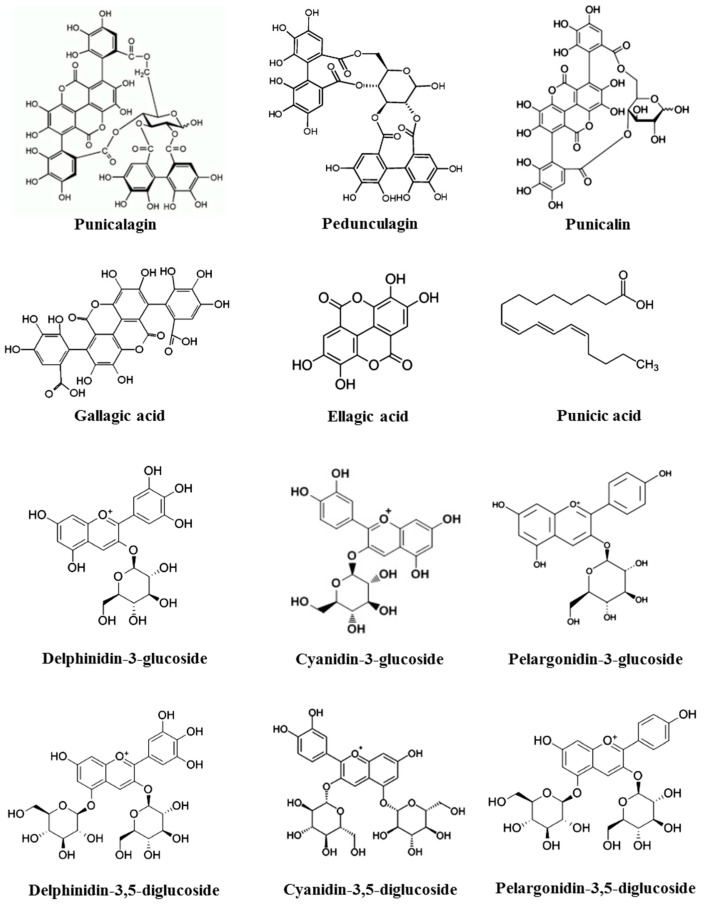
Chemical structures of major constituents present in pomegranate.

The pomegranate fruit consists of white to deep purple seeds embedded in a white spongy astringent membrane surrounded by a thick reddish skin, or pericarp. Pericarp constitutes almost 50% of the fruit weight and is a rich source of bioactive constituents, such as phenolics, flavonoids, ellagitannins, and proanthocyanidin compounds. It also contains various minerals, mainly potassium (K), nitrogen (N), calcium (Ca), phosphorus (P), magnesium (Mg), and sodium (Na), as well as complex polysaccharides. The remaining 50% of the fruit consists of seeds (constituting 10% of the fruit weight) and arils (constituting 40% of the fruit weight) [17]. Pomegranate seeds hold strong antioxidant and anti-inflammatory properties due to the high content of hydrolysable tannins (punicalagin, pedunculagin, punicalin, gallagic acid, ellagic acid, and esters of glucose) and anthocyanins (delphinidin-3-glucoside, cyanidin-3-glucoside, delphinidin-3,5-diglucoside, cyanidin-3,5-diglucoside, pelargonidin-3,5-diglucoside, and pelargonidin-3-glucoside) (Figure 1) [34,62]. Various organic acids, such as ascorbic acid, citric acid, and malic acid, etc., are also reported to be present in the seed coat [17], while the arils contain water (85%), sugars (10%), mainly fructose and glucose, and pectin (1.5%). Arils are a rich source of bioactive compounds such as phenolics and flavonoids, principally anthocyanins. Pomegranate seed oil consists of mainly conjugated linolenic acid. Interestingly, punicic acid, a conjugated isomer of linolenic acid found uniquely in pomegranate oil, constitutes 70%–76% of the seed oil [63]. Sterols, steroids, and cerebroside, a key component of mammalian myelin sheaths, constitute the minor share of the seed oil [64].

Pomegranate leaves contain some unique tannins in addition to containing glycosides of apigenin, which is flavone with progestinic and anxiolytic characters [65,66]. Leaves also represent a rich source of elements such as N, K, Ca, and Fe, with levels of elements varying with the season and the stage and maturity of the plant [24]. For example, K content is reported to be high in young leaves, while levels of Ca and Fe are considered to be highest in old leaves. Medium-age plants have high N content. The content of N, however, is reduced at the time of flowering and setting of the fruit. N content is further reported to decline with the fruit maturity [67,68].

## 3. Pomegranate and Skin Cancer

Skin cancer is the most common form of cancer in fair skinned individuals. It is estimated that, in the United States alone, nearly 83,510 new skin cancer cases will be diagnosed in the year 2016 that will result in nearly 13,650 deaths [5]. Considering this grim statistics, it is essential to develop novel and effective skin cancer chemopreventive/chemotherapeutic strategies. Sun exposure is the major known environmental factor influencing the development of skin cancer of all types. Ultraviolet B (UVB) radiation coming from the sun represents a major risk factor for the development of skin cancer [69,70]. At the molecular level, exposure of skin to UVB radiation leads to activation of multiple signaling pathways in the skin. These pathways control DNA damage repair, oxidative balance, inflammation, immune responsiveness, and cell survival or cell death [69,70]. Pomegranate fruit extract (PFE), pomegranate juice (PJ), and pomegranate seed oil (PSO) have been tested in cell culture, reconstituted human skin models, and animal models of skin cancer and exhibit immense potential for preventing UVB-induced skin cancer.

PFE was shown to inhibit UVB-induced phosphorylation of the mitogen-activated protein kinases (MAPK) in normal human epidermal keratinocytes (NHEK) [25]. Pretreatment of NHEK with PFE resulted in a dose- and time-dependent inhibition of UVB-induced phosphorylation of ERKl/2, JNK1/2, and p38 proteins. PFE was also found to inhibit UVB-mediated activation of the nuclear factor kappa B (NFκB) pathway, an effect that was accompanied with reduced phosphorylation of IκBα, increased stabilization of IκBα protein, and reduced activation of IKKα protein [25]. The photo-protective effects of PFE extend against the harmful effects of UVA radiation as well, shown in a study wherein PFE was evaluated for its effects against UVA-mediated activation of signal transducer and activator of transcription 3 (STAT3), AKT, and ERK1/2 in NHEK [29]. While UVA was shown to result in increased phosphorylation of STAT3, AKT, mTOR, and ERK1/2 in NHEK, pretreatment with PFE resulted in inhibition of these events in a dose-dependent manner. Interestingly, PFE treatment to NHEK resulted in a significant inhibition of UVA-induced expression in Ki-67 and PCNA, and it also led to an enhanced expression of pro-apoptotic Bax and Bad with downregulation of antiapoptotic Bcl-xL protein [29]. Similarly, polyphenol-enriched pomegranate extract (POMx) was evaluated for its effect on UVB-mediated oxidative stress and markers of photoaging in immortalized human HaCaT keratinocytes, and it was found that pretreatment protects the cells from UVB-induced oxidative stress and markers for photoaging [26]. Treatment of HaCaT cells with POMx prior to UVB irradiation resulted in inhibition of UVB-mediated decrease in glutathione content as well as UVB-induced lipid peroxidation [26]. POMx treatment was also found to protect HaCaT cells against UVB-induced photoaging as evidenced by reduction in expression of UVB-induced upregulation of matrix metalloproteinases (MMPs) (such as MMP-1, -2, -7, and -9) and phosphorylation of MAPK [26]. Another recent study carried out on HaCaT cells evaluated photoprotective effects of a nanoemulsion of PSO against UVB radiation and found that PSO protected cells against UVB-induced DNA damage in a dose-dependent manner [30]. Similarly, PFE was shown to protect human fibroblast cells from the UVA- and UVB-induced damage by reducing activation of NFκB, downregulating active caspase 3, and increasing cells in G0/G1 phase [31]. Pomegranate-derived products, such as PJ, POMx, and PSO, were tested for their UVB protective effects in reconstituted human skin [32]. Pretreatment of EpiDerm with pomegranate-derived products inhibited UVB-induced DNA damage as well as activation of MMPs in the EpiDerm, thus indicating the usefulness of pomegranate-derived products against UVB-induced damage to human skin [32].

The chemopreventive properties of PFE were further evaluated in mice exposed to UVB radiation. Afaq et al. [33] evaluated the effects of PFE administration via drinking water against the early biomarkers of UVB-induced skin cancer in SKH-1 hairless mice that were exposed to a single dose of UVB (180 mJ/cm^2^) irradiation. It was observed that PFE treatment augmented UVB-mediated increase in the protein expression of p21 and p53, but also resulted in inhibition of NFκB signaling as evidenced by reduced nuclear translocation of NFκB, reduced activation of IKKα, as well as decreased phosphorylation and degradation of IκBα. Photochemopreventive effects of PFE administered via drinking water were further evaluated in mice exposed to multiple UVB irradiations [27]. Oral administration of PFE inhibited UVB-induced epidermal hyperplasia, leukocyte infiltration, and protein oxidation. Oral administration of PFE also attenuated UVB-induced activation of key inflammatory and cell proliferative pathways such as NFκB and MAPK. Reduction in UVB-induced protein expression of COX-2, iNOS, PCNA, cyclin D1, and MMPs in mouse skin further supported anti-inflammatory and anti-proliferative effects of PFE [27]. More importantly, oral administration of PFE in drinking water reduced UVB-induced skin tumor incidence and tumor multiplicity in SKH-1 hairless mice [28]. PFE treatment resulted in inhibition of UVB-induced phosphorylation of STAT3, and NFκB/p65 with a concomitant decrease in the protein expressions of iNOS, and COX-2 in uninvolved skin from tumor-bearing mice and skin tumors compared to non-PFE-treated animals. These data suggest that PFE protects against UVB-induced skin tumorigenesis, at least in part, by modulating transcription factors STAT3 and NFκB.

PFE’s capability to inhibit skin cancer was also determined in 7,12-dimethylbenz(a)anthracene (DMBA) initiated and 12-*O*-tetradecanoylphorbol-3-acetate (TPA) promoted chemical carcinogenesis model. Topical application of PFE to mouse skin resulted in delayed onset of skin tumor formation, as well as a significant reduction in tumor incidence and tumor burden in mice [34]. PFE was found to inhibit TPA-induced skin edema, thus highlighting PFE’s anti-inflammatory effects. It was further observed that topical application of PFE inhibited TPA-induced activation of NFκB and IKKα, phosphorylation and degradation of IκBα, as well as phosphorylation of ERK1/2, p38 and JNK1/2. Hora et al. [35] also demonstrated the anti-skin tumorigenic effect of PSO by using chemical carcinogenesis protocol (DMBA initiated and TPA promoted) in CD-1 mice. A significant reduction in tumor incidence and tumor multiplicity was observed in PSO-treated mice compared to the untreated mice. To further improve the anticancer effects of PFE, George et al. [71] carried out a combinatorial phytochemical treatment approach and administered PFE and diallyl sulfide (DAS), alone and in combination in chemical carcinogenesis model. It was observed that PFE and DAS exerted inhibition of tumor development synergistically. While PFE and DAS reduced tumor incidence by ~55% and ~45%, respectively, even more potent reduction (~84%) of tumor incidence was observed in mice that received both PFE and DAS. These data suggest that PFE and PSO exhibit chemopreventive effects against skin tumorigenesis.

## 4. Pomegranate and Breast Cancer

Breast cancer is the second leading cause of cancer-related deaths in women. In 2016, an estimated 246,660 new cases of invasive breast cancer are expected to be diagnosed in women in the Unites States, along with 61,000 new cases of non-invasive (in situ) breast cancer [5]. Old age, family history of breast cancer, early age at menarche, late age of menopause, long-term use of estrogen-replacement therapy, and later age at birth of first-born child are some of the common established risk factors for the development of breast cancer. Steroid hormones, particularly estrogens, are believed to play a central role in development of breast cancer [72]. In recent years, the link between dietary factors and breast cancer risk has been a significant area of research. Studies have shown the beneficial effects of pomegranates in breast cancer [73]. Kim et al. [74] reported that polyphenols from fermented PJ, pericarp, and PSO inhibited aromatase, which converts androgen to estrogen and plays an important role in breast carcinogenesis. It was shown that polyphenols derived from fermented PJ, pericarp, and PSO were also able to inhibit 17-β-hydroxysteroid dehydrogenase, an estrogen biosynthetic enzyme. Consistent with their anti-estrogenic effects, polyphenols from fermented PJ and pericarp exerted a cell growth inhibitory effect against both MCF-7 and MB-MDA-231 breast cancer cell lines. Polyphenols from fermented PJ also inhibited DMBA-induced cancerous lesion formation in a murine mammary gland organ culture [74]. Another study revealed the potential of pomegranate ellagitannins-derived compounds exhibiting anti-proliferative and anti-aromatase activities in breast cancer cells [36]. The pomegranate ellagitannin-derived compounds including ellagic acid, gallagic acid, and urolithins A and B (acetylated, methylated, and sulfated analogues) were investigated for their anti-aromatase activity by using placental microsome aromatase assay and a live cell based assay. It was observed that urolithin A, methylated urolithin A, urolithin B, methylated urolithin B, acetylated urolithin B, urolithin B sulfate, and gallagic acid significantly inhibited aromatase activity in the placental microsomes. When these active compounds were further compared in an aromatase over-expressing cell line (MCF-7aro), urolithin B was found to be the most potent aromatase inhibitor. It significantly inhibited aromatase activity at 2.35 μM (*p* ≤ 0.05) and 4.7 μM (*p* ≤ 0.01). Gallagic acid was observed to exhibit anti-aromatase activity as well; it inhibited aromatase activity significantly at a dose of 4.7 μM (*p* ≤ 0.01). Urolithins were further tested for their effects against testosterone-induced cell proliferation, and it was found that urolithin B inhibits testosterone-induced cell proliferation. Urolithin B was followed by gallagic acid for the anti-proliferative effects [36]. These data suggest that intake of pomegranate may be a beneficial strategy for breast cancer chemoprevention.

The methanolic extract of pomegranate pericarp (PME) was shown to possess a selective estrogen receptor modulator (SERM) property in human breast cancer cell lines and in vivo models of estrogen deprivation [37]. SERMs are ligands for the estrogen receptor (ER) and may exert an agonist or an antagonist function depending on the type of tissue. SERMs are frequently used for the therapy of estrogen-dependent breast cancers. PME treatment led to significant dose-dependent inhibition of cell growth in MCF-7 cell line that are ER^+^, while there was no effect on the proliferation of ER^−^ MDA MB-231 cells. PME also inhibited 17β-estradiol-induced proliferation in MCF-7 cells. In addition, PME was found to downregulate the expression of estrogen responsive genes such as ERα, pS2, and PR in the MCF-7 cells. Finally, the lack of esterogenicity of PME was confirmed in ovariectomized (OVX) mice, wherein uterine wet weights and epithelial heights were assessed as markers of esterogenicity. It was observed that while 17β-estradiol increased absolute and normalized uterine wet weight in OVX animals by approximately two times, there was no significant difference in weight of uterus between the groups that received PME and the vehicle-treated OVX control group, indicating the lack of estrogenecity of PME on uterine endometrium. Similarly, from the uterine histology it was clear that while 17β-estradiol induced proliferation of uterine epithelium, there was no luminal epithelial proliferation in PME treated OVX mice [37]. Further, Rocha et al. [38] tested PJ and its components for their effects on a number of precarious processes involved in breast cancer metastasis. They used two breast cancer cell lines, MDA-MB-231 cells (ER^−^) and MCF-7 (ER^+^), and the non-neoplastic cell line MCF10A, and showed that PJ, or a combination of its components, luteolin plus ellagic acid plus punicic acid, increased cancer cell adhesion, decreased cancer cell migration, and reduced growth of the breast cancer cells, without affecting the normal cells. PJ and the three components also prevented the production of pro-inflammatory cytokines/chemokines in the cancer cells. Interestingly, the study also revealed that PJ and its components promoted expression of genes involved in increased adhesion, inhibited cell migration stimulating genes, and prevented chemotaxis of the cancer cells to stromal cell-derived factor 1α.

Several studies investigating the chemopreventative potential of pomegranate against breast cancer have highlighted the importance of pro-apoptotic and antioxidant properties manifested in the PFE and its components [6,14,73]. Punicic acid, a polyunsaturated fatty acid found in PSO, was reported to significantly inhibit growth, as well as induce apoptosis of estrogen sensitive and insensitive breast cancer cell lines, namely MDA-MB-231 and MDA-ER-7 cells [39]. Methanolic extract of PFE was shown to reduce proliferation of MCF-7 breast cancer cells while increasing the number of apoptotic cells in a dose-dependent manner [40]. These effects of PFE were associated with an increased expression of pro-apoptotic gene Bax, and a reduced expression of anti-apoptotic gene Bcl2. Costantini et al. [41] identified punicic acid and its congeners as the most abundant compounds of the hydrophilic fraction (80% aqueous methanol extract) from PSO and evaluated their possible anti-inflammatory effects on breast cancer lines (MCF-7 and MDA-MB-231). The study indicated that the hydrophilic extract treatment resulted in a significant decrease in cell viability in both breast cancer cell lines with an increase in G0/G1 phase of the cell cycle compared to untreated cells and with no significant increase in apoptosis in these two breast cancer cell lines. This study also indicated that with increasing amounts of the hydrophilic extracts of PSO, there was a decrease in the levels of VEGF and pro-inflammatory cytokines (IL-2, IL-6, IL-12, IL-17, CXCL10, MIP-1α, MIP-1β, MCP-1, and TNF-α).

More recently, a study examining the anti-breast cancer properties of PFE has focused on gene expression changes that occur at the whole genome level in the MCF-7 cells [42]. It was observed that the reduced proliferation of MCF-7 cells by PFE treatment led to differential expression of 903 genes, of which 505 genes were upregulated, while 398 genes were downregulated. A majority of the genes that were upregulated were involved in regulation of apoptosis, while the genes that were downregulated included genes involved in mitosis, chromosomal organization, RNA processing, DNA damage response, and DNA repair. Genes such as *MRE11*, *RAD50*, *NBS1*, *RAD51*, *BRCA1*, *BRCA2*, *BRCC3*, and *MSH6* that are involved in DNA damage response and repair were found to be downregulated [42]. Another cDNA microarray based study for understanding the molecular mechanisms underlying the ellagic acid-induced growth inhibition on MCF-7 cells proposes that ellagic acid inhibits the growth of breast cancer cells by cell cycle arrest and inhibition of proliferation [43]. It was observed that changes in genes that belong to TGF-β/Smads signaling pathway as a molecular mechanism of ellagic acid regulated cell cycle arrest in MCF-7 cells. TGF-β is known to be a strong tumor suppressor that promotes cell growth inhibition, apoptosis, and differentiation [75,76].

Studies evaluating the chemopreventive effect of orally administered pomegranate emulsion (PE) against breast cancer were performed in DMBA-induced mammary tumorigenesis in female Sprague-Dawley rats [77]. Rats that were administered PE exhibited reduction in both tumor incidence and cumulative tumor burden compared to control rats. PE-treated tumors exhibited almost normal ductal and alveolar structure with uniform epithelial cells without any sign of hyperplasia when compared with tumors from control rats that showed extensive epithelial proliferation histologically. PE exerted its chemopreventive effect against DMBA-initiated mammary tumors by reducing cell proliferation and inducing apoptosis [77]. Mechanistic information underlying the chemopreventive effects of PE was further evaluated in another study from the same group showing that PE-treated tumors showed reduced expression of ER-α and ER-β, as well as reduced expression, cytoplasmic accumulation, and nuclear translocation of β-catenin [44]. These data suggest that PE-induced disruption of ER and Wnt/β-catenin signaling pathways is the molecular basis of its chemopreventive effect against DMBA-inflicted rat mammary tumorigenesis.

## 5. Pomegranate and Prostate Cancer

Prostate cancer (PCa) is the second major cause of cancer related deaths in men in the United States. The latest count for new PCa diagnoses is estimated to be 180,890 with 26,120 estimated death cases in United States [5]. Pomegranate has been shown to exhibit beneficial effects against PCa in cell culture and animal studies. Lansky et al. [78] reported that ellagic acid, caffeic acid, luteolin, and punicic acid that are found in substantial amounts in the peels, PJ, and PSO of the pomegranate fruit reduced the invasive potential of PC-3 cells. A supradditive inhibition in PC-3 cell invasion was observed when caffeic acid, luteolin, and punicic acid were equally combined at the same gross dose when compared to individual agents. Albrecht et al. [79] examined the effects of pomegranate-derived fractions, namely pomegranate pericarp polyphenols, fermented PJ polyphenols, and cold-pressed PSO on PCa growth, apoptosis, invasion, and tumor growth. Treatment of human PCa cells with PSO, fermented PJ polyphenols, and pomegranate pericarp polyphenols reduced cell proliferation, increased cells in G2/M phase, and induced apoptosis. Pomegranate-derived fractions treatment reduced PC-3 invasion and also inhibited tumor growth in athymic mice implanted with PC-3 cells. Malik et al. [45] reported that the modulation of cdk is the key mechanism involved in the pro-apoptotic and anti-proliferative effects of PFE. Treatment of highly aggressive PC-3 cells with PFE resulted in a cell growth inhibition and induction of apoptosis. The study documented that PFE essentially downregulated cyclins D1, D2, E, cdk2, cdk4, and cdk6 and upregulated p21 and p27. PFE-induced apoptosis in PC-3 cells was accompanied with an increase in cleaved PARP, a decrease in Bcl-2, and a concomitant increase in Bax. Additionally, oral administration of PFE in drinking water to athymic nude mice implanted with CWR22Rν1 cells resulted in a significant inhibition in tumor growth that was associated with a reduction in the secretion of prostate-specific antigen (PSA) in the serum [45]. Treatment of LNCaP cells with ellagic acid, a component of PJ, induced apoptosis by increasing the Bax/Bcl-2 ratio and cleavage of caspase 3. Ellagic acid treatment increased the expression of p21 and p27, whereas expression of cyclin D1 and cdk1 was decreased [80]. These data indicate that ellagic acid is a potential chemotherapeutic agent against PCa.

Studies have demonstrated that PFE exhibits beneficial effects by reducing proliferation and inducing apoptosis in PCa cells by targeting multiple signaling pathways. Treatment of human metastatic castration-resistant PCa cells with POMx induced cell death by reducing the expression of survivin and inhibiting STAT3. In this study, POMx treatment also enhanced the efficacy of docetaxel in reducing C4-2 tumor growth in athymic nude mice [46]. Oral administration of PJ in drinking water to transgenic rats for adenocarcinoma of the prostate resulted in a decrease in the incidence of adenocarcinoma in the lateral prostate as compared to the control group. Ellagic acid also reduced the progression of prostatic lesions or adenocarcinoma in lateral prostate. Both PJ and ellagic acid suppressed prostate carcinogenesis by activation of caspase 3-mediated apoptosis. Insulin-like growth factor-1 (IGF-1) is upregulated in several cancers including PCa, and exhibits mitogenic and anti-apoptotic effects. IGF binding protein (IGFBP)-3 is the most abundant of the IGFBPs and it binds to IGF-1 and regulates the availability and ligand function of IGF-1 to IGF-1 receptor [81]. Administration of PFE in drinking water to TRAMP mice inhibited prostate carcinogenesis by downregulating IGF-1/Akt/mTOR pathways [49]. Treatment of LAPC4 PCa cells with POMx resulted in the inhibition of cell proliferation and induction of apoptosis. Co-treatment of LAPC4 cells with POMx and IGFBP-3 resulted in the additive inhibition of cell growth and synergistic activation of apoptosis. In addition, co-treatment with IGF-1 and POMx blocked apoptosis in 22Rν1 cells induced by POMx. However, the effects of IGF-1 in inhibiting POMx-induced apoptosis was abolished in IGF-1 receptor null MEF cells, indicating the significance of the IGF1 receptor in antagonizing the effects of POMx [50]. PFE treatment of androgen independent DU145 cells with constitutive activation of NFκB resulted in inhibition of cell proliferation and induction of apoptosis by blockade of NFκB. In addition, PFE treatment inhibited the growth of androgen-sensitive and androgen-independent PCa that lack basal NFκB activity. These data suggest that PFE inhibits the growth of PCa cells in NFκB-dependent and -independent manner. Dietary supplementation of PFE to castrated immunodeficient mice delayed the emergence of LAPC4 androgen-independent xenografts by inhibition of NFκB activity [47]. A proteomics study evaluating the effects of PJ on DU145 cells demonstrated that PJ potentially limits PCa by modulating the expression of genes associated with apoptosis, the NFκB signaling pathway, invasion/metastasis, angiogenesis, and cytoskeleton [48].

Androgens and their receptors are crucial factors contributing to PCa development, growth, and progression [82]. Treatment of androgen-dependent (LNCaP) and androgen-independent (LNCaP-AR and DU145) human PCa cell lines with PFE and PJ displayed decreased expression of genes involved in androgen biosynthesis, such as 3β-hydroxysteroid dehydrogenase type 2, aldo-keto reductase family 1 member C3, steroid 5α reductase type 1, and AR [51]. These findings suggest that the polyphenols present in pomegranate may be useful in androgen-independent PCa and in subsets of PCa where there is up-regulation of AR. The cytochrome P450 (CYP) proteins are responsible for bioactivation of xenobiotics and endobiotics. The CYP1 isoforms activate a number of polycyclic aromatic hydrocarbons to exert their detrimental effects. Studies have shown that CYP1B1 plays an important role in the initiation and promotion of cancer and, therefore, represents an attractive target for cancer chemoprevention. It was observed that systemically available metabolites of PJ could effectively inhibit enzyme activity/expression of CYP1B1 [52]. Previous studies have shown that polymorphisms in CYP1B1 and PSA genes increased the risk of PCa [83]. Therefore, these studies suggest that consumption of PJ may reduce the incidence of PCa. A significant amount of chemopreventive studies explicitly suggest that the potential protective effect of PJ against PCa is largely attributed to ellagitanins, representing the most abundant polyphenols present in PJ. In this context, the main metabolite to concentrate in the human prostate gland upon consumption of PJ was urolithin A glucuronide, (3,8-dihydroxy-6*H*-dibenzo[*b*,*d*]pyran-6-one glucuronide), together with traces of urolithin B glucuronide, (3-hydroxy-6*H*-dibenzo[*b*,*d*]pyran-6-one glucuronide) and dimethyl ellagic acid [84]. These data indicate urolithin glucuronides and dimethyl ellagic acid may be the bioactive metabolites accounting for the chemopreventive effects of PJ against PCa.

Pantuck et al. [85] performed the first clinical trial of PJ in PCa patients following surgery and radiation. The study reported that oral consumption of PJ had no adverse effects and significantly increased PSA doubling times (PSADT) in men with PCa. A randomized, multi-center, double-blind phase II study was performed to determine the biological activity of two doses of POMx in PCa patients by monitoring PSADT following initial therapy for localized PCa. Treatment of PCa patients with POMx increased the PSADT by almost six months in both the treatment arms [86]. To determine the effects of PFE treatment on PSADT in PCa patients with rising PSA after primary therapy, a randomized, double-blind, placebo-controlled study was performed. It was observed that PFE did not significantly prolong the PSADT in patients with rising PSA after primary therapy compared to the placebo-treated group. In addition, this study indicated that patients with MnSOD AA genotype receiving PFE may be more sensitive in prolonging PSADT [87]. A phase II, randomized double-blind trial of men with PCa undergoing radical prostatectomy showed that there was no significant reduction in the level of 8-hydroxy-2′-deoxyguanosine in POMx treated group compared to the placebo-treated group. In addition, there were no differences in expression of pS6, NFκB, or Ki67 within PCa tissues between arms [88]. Stenner-Liewen et al. [89] evaluated the therapeutic impact of PJ as an adjunct intervention in a cohort of more advanced or metastatic PCa, of which 68% had castration-resistant PCa. The patients continued their baseline treatment, such as androgen deprivation therapy. The study concluded that consumption of PJ did not result in a significant decline in PSA levels compared to placebo.

## 6. Pomegranate and Lung Cancer

Lung cancer is the leading cause of cancer-related mortality worldwide. According to statistics, an estimated 224,390 new cases and 158,080 deaths are expected to be caused by lung and bronchus tumors in both sexes in the United States in 2016 [5]. Current research has documented the potential of PFE in inhibiting the growth of lung cancer cells in culture. PFE treatment resulted in a substantial decrease in the viability of human lung carcinoma A549 cells but had minimal effects on normal human bronchial epithelial cells. PFE treated A549 cells displayed a dose-dependent arrest of cells in the G0/G1 phase of the cell cycle, which was linked to induction of WAF1/p21 and KIP1/p27 and a decrease in the expression of cyclins and cdks. Furthermore, PFE treatment inhibited several signaling pathways, including MAPK, PI3K/AKT, and NFκB [53]. Using punicalagin isolated from the pomegranate husk, Aqil et al. [90] showed that punicalagin possesses strong antioxidant activity by decreasing the accumulation of oxidative DNA products and displays strong anti-proliferative activity against lung cancer cells. Punicalagin and ellagic acid, the major constituents of the pomegranate peel, were shown to possess strong anti-proliferative activities. Both A549 and H1299 lung cancer cell lines displayed comparable levels of sensitivity to the tested compounds [54]. A recent study evaluated anti-proliferative properties of pomegranate peel against different cancer cells including lung cancer. This study indicated that the anti-proliferative properties of pomegranate are not solely confined to the edible part of the pomegranate fruit [91]. Another study showed that pomegranate leaf extract (PLE) reduced cell proliferation of non-small cell lung carcinoma cell lines (A549, H1299) and mouse Lewis lung carcinoma cell line LL/2. PLE treatment reduced H1299 cell migration and invasion, indicating usefulness of the PLE in reducing metastasis [92].

The chemopreventive efficacy of PFE was evaluated using benzo(a)pyrene [B(a)P] and *N*-nitroso-tris-chloroethylurea (NTCU) induced lung tumor models of A/J mice. It was found that compared to the control mice that were exposed to B(a)P and NTCU, mice that received PFE in drinking water had statistically significant lower lung tumor multiplicities. PFE-treated mice showed decreased activation of NFκB, MAPK, and PI3K pathways leading to reduced cell proliferation and angiogenesis in lungs of B(a)P- and NTCU-treated mice [55]. Another study revealed that oral consumption of PFE in drinking water reduced tumor growth in athymic nude mice implanted with A549 cells [53]. Punicalagin and ellagic acid were shown to possess strong anti-mutagenic and anti-proliferative activities in B(a)P-induced lung cancer model [54]. The results from these studies suggest the usefulness of PFE as a chemopreventive/chemotherapeutic agent against human lung cancer. Pomegranate peel aqueous extract was evaluated for the antioxidant and anti-inflammatory properties and it was found that it inhibited neutrophil myeloperoxidase (MPO) activity. Although it showed no effect on superoxide generation, it attenuated lipopolysaccharide-induced lung inflammation in mice. Inhibition of MPO activity by pomegranate peel aqueous extract could be attributed to its anti-inflammatory action [56]. Similarly, Husari et al. [57] tested the antioxidant activity of PJ in response to hyperoxia and observed that rats exposed to hyperoxia displayed increased ROS production and increased levels of pro-inflammatory cytokines (IL-1β and IL-6) in the lungs. Administration of PJ in drinking water resulted in significant attenuation of these effects of hyperoxia, thus indicating that PJ possesses strong anti-inflammatory activities besides possessing strong antioxidant properties. Recently it has been shown that PFE possesses strong antioxidant activity in methotrexate-treated rats. Methotrexate-treated rats exhibited a significant increase in malondialdehyde levels, total oxidant status, and oxidative stress index in the serum and lung; however, pretreatment of rats with PFE reversed these effects [58].

## 7. Pomegranate and Colon Cancer

Colorectal cancer is the third most commonly diagnosed cancer and the third leading cause of cancer deaths in both men and women in the United States. According to the American Cancer Society’s most current estimates, approximately 134,490 new cases of colorectal cancer will be diagnosed in the United States in 2016 [5]. Increasing evidence supports that regular consumption of fruits, vegetables, and grains that are rich in polyphenols may reduce the risk of colon cancer [93,94,95]. Pomegranates have also been studied for their protective effects against colon cancer. Seeram et al. [96] studied the effect of PJ and its purified polyphenols on human colon cancer cell lines (HT-29, HCT116, SW480, SW620), and found that PJ displayed the highest anti-proliferative and pro-apoptotic effects compared to its purified polyphenols. Thus, this study suggests that separation of individual polyphenols from PJ may decrease the overall anti-proliferative activity, owing to the requirement of multiple compounds for chemical synergy and multifactorial effects compared to single purified agents. Treatment of HT-29 cancer cells with PJ inhibited TNFα-induced COX-2 protein expression and also suppressed NFκB DNA binding and AKT activity [59]. These studies indicate that PJ plays an important role in downregulating inflammatory signaling pathways in colon cancer cells. Larrosa et al. [97] studied the induction of apoptosis in Caco-2 colon cancer cells by punicalagin and ellagic acid from PJ. Their study revealed that treatment of Caco-2 cells with these agents resulted in the release of mitochondrial cytochrome c into the cytosol, activation of caspase-3 and -9, and down-regulation of anti-apoptotic Bcl-xL. Both punicalagin and ellagic acid treated Caco-2 cells resulted in decreased protein expression of cyclins, as well as arrest of cells in S phase of the cell-cycle. The authors suggest that the anti-carcinogenic effect of pomegranate ellagitannins could largely be due to their hydrolysis product ellagic acid, which induced apoptosis in colon cancer cells.

The consumption of PSO rich in conjugated linolenic acid in the diet was found to significantly inhibit the growth of azoxymethane-induced colonic adenocarcinomas in male F344 rats without causing any adverse effects [64]. Furthermore, the dietary intake of PSO deceased the multiplicity of colonic carcinomas in the colon of rats. Additionally, PJ was studied for the colon cancer chemopreventive effects in vivo on azoxymethane (AOM)-induced aberrant crypt foci in Fisher 344 male rats that were administered 20% PJ before and after treatment with azoxymethane [60]. The histopathology of the rat colon studied after the 17th week of treatment revealed a significant decrease in the number of large crypts in PJ-fed rats. The protective effect of the PJ was also evident from the PJ-fed rats’ increased food intake and weight gain. Further, the activity of hepatic glutathione S-transferase was significantly higher in rats fed with PJ, which probably supports the mechanism of pomegranate chemopreventive activities. These results suggest favorable effects of pomegranate against the development of colonic tumors in rats.

Earlier studies have shown that urolithins with potential cancer chemopreventive effects can reach high concentrations in the colon of animals following ellagitannins intake [98]. Recently, an interesting study based on the pilot trial with colorectal cancer (CRC) patients that needed surgical resection of colon tissues revealed significant levels of ellagic acid derivatives and urolithins found in human colon tissues from CRC patients after consumption of pomegranate extract (PE) [99]. The study was completed by twenty-six patients assigned in two PE groups: PEs with low (PE-1) and high (PE-2) punicalagin:ellagic acid ratios. Both PEs were well tolerated by the patients with no adverse effects, such as dyspepsia, allergic reactions, etc., reported during the study. Free ellagic acid, various ellagic acid conjugates, as well as gallic acid, and up to 12 different urolithins, were found in colon tissues, but no ellagitannins were found. The normal colon tissues had higher levels of individual and total metabolites than in malignant colon tissues. The main urolithins produced were urolithin A or isourolithin A (54% and 46% patients with urolithin A or isourolithin A phenotype). The study found higher urolithin formation after intake of the extract with higher free ellagic acid. Additional studies based on a randomized, double-blind, controlled trial with 35 CRC patients consuming 900 mg PE daily before surgery and control (no PE intake, *n* = 10) CRC patients, found an in vivo specific modulatory effect of the intake of PEs enriched in ellagic acid or ellagitannins (punicalagin) on specific miRs (miR-646, miR-1249, miR-135b-5p, miR-135b-3p,miR-92b-5p, miR-765, miR-496, miR-181c-3p, and miR-18a-3p) in human malignant and normal colon tissues from CRC patients [61]. The data revealed no direct relationship between the observed changes in expression levels of specific miRs and the presence of ellagic acid derivatives and urolithins in human colon tissues. Although a preliminary in silico analysis showed a potential involvement of the modulated miRs with cancer-related genes and pathways, the significance of these results in relation to cancer prevention is yet to be understood, warranting further research.

## 8. Conclusions

In the current setting, cancer prevention via dietary agents is quite a promising arena of oncology that has drawn a significant amount of attention from both scientists in basic and clinical sciences and the general masses due to dietary agents’ proven ability to prevent or suppress cancers, their low cost, and easy availability. However, current challenges relate to establishing the key component of these dietetic agents responsible for the anticancer effects and the mechanisms through which they suppress cancer. Accumulating research provides extensive evidence related to biological activities of pomegranate-derived products particularly with respect to their anticancer properties. Studies have suggested the whole pomegranate fruit, as well as its juice and oil, as promising chemopreventive/chemotherapeutic agents, as they exert anti-inflammatory, anti-proliferative, and anti-tumorigenic effects by modulating multiple signaling pathways. More in vitro and in vivo studies are needed to assess the combinatorial effect of pomegranate with other compounds to determine whether additive or synergistic, or even antagonistic, effects are observed. Considerable data demonstrates the in vitro and in vivo efficacy of pomegranate against cancer growth and promotion; however, well-designed human clinical trials are necessary to validate the usefulness of these natural agents either alone or in combination with current therapy for the prevention and treatment of skin, breast, prostate, lung, and colon cancers.

## Figures and Tables

**Table 1:** Molecular targets of pomegranate in cancers.

Cancers	Molecular Mechanism(s)/Cellular Targets	References
**Skin**	Inhibits UVB-mediated activation of MAPK, NFκB and STAT3 signaling pathways	[25,26,27,28]
	Inhibits UVA-mediated phosphorylation of STAT3, AKT, ERK1/2, mTOR and p70S6K Decreases UVA-mediated upregulation of PCNA and Ki-67 expression Up-regulates UVA-mediated Bax and Bad expression	[29]
	Inhibits UVB-mediated decrease in GSH Inhibits UVB-mediated up-regulation of MMPs-1,-2,-7 and -9	[26]
	Inhibits UVB-induced DNA damage and NFκB activation	[30,31]
	Inhibits UVB-induced DNA damage Inhibits UVB-induced MMP-2 and -9 activities Decreases UVB-induced MMPs-2,-3,-9 expression Inhibits UVB-induced c-Jun phosphorylation and tropoelastin protein expression Reduces UVB-mediated PCNA, ODC and COX-2 expression Augments UVB-mediated increase in p53 and p21 expression	[27,32,33]
	Inhibits TPA-mediated increase in epidermal ODC activity and COX-2 expression Inhibits TPA-induced MAPK phosphorylation and NFκB activation	[34,35]
**Breast**	Exhibits anti-estrogenic and anti-aromatase activities	[36]
	Downregulates estrogen responsive genes	[37]
	Reduces VEGF and pro-inflammatory cytokines/chemokines	[38,39,40,41]
	Downregulates expression of genes involved in DNA damage response and repair	[42]
	Regulates TGF-β/Smads pathway	[43]
	Disrupts ER and Wnt/β-catenin signaling pathways	[44]
**Prostate**	Decreases serum PSA levels	[45]
	Inhibits STAT3 phosphorylation and NFκB activation	[46,47,48]
	Inhibits IGF-1/AKT/mTOR signaling	[49,50]
	Inhibits androgen biosynthesis enzymes such as 5α-reductase type I and 3β-hydroxysteroid dehydrogenase type II	[51]
	Inhibits CYP1B enzyme activity/expression	[52]
**Lung**	Increases p21 and p27 protein expression Downregulates cyclins/cdks, PCNA and Ki-67 expression Inhibits MAPK, PI3K/AKT and NFκB signaling pathways	[53]
	Inhibits DNA adduct formation	[54]
	Decreases markers of proliferation, inflammation and angiogenesis Inhibits phosphorylation of MAPK and c-Met	[55,56,57]
	Decreases lipid peroxidation and increases total antioxidant capacity levels	[58]
**Colon**	Inhibits COX-2 expression, AKT phosphorylation and NFκB DNA binding activity	[59]
	Increases hepatic GST activity	[60]
	Modulates miR-646, miR-1249, miR-135b-5p, miR-135b-3p, miR-92b-5p, miR-765, miR-496, miR-181c-3p and miR-18a-3p	[61]
